# Cystatin SN inhibits auranofin-induced cell death by autophagic induction and ROS regulation via glutathione reductase activity in colorectal cancer

**DOI:** 10.1038/cddis.2017.100

**Published:** 2017-03-16

**Authors:** Byung Moo Oh, Seon-Jin Lee, Hee Jun Cho, Yun Sun Park, Jong-Tae Kim, Suk Ran Yoon, Sang Chul Lee, Jong-Seok Lim, Bo-Yeon Kim, Yong-Kyung Choe, Hee Gu Lee

**Affiliations:** 1 Immunotherapy Convergence Research Center, Korea Research Institute of Bioscience and Biotechnology, Daejeon, Republic of Korea; 2 Department of Biomolecular Science, University of Science and Technology (UST), Daejeon, Republic of Korea; 3 Department of Biological Sciences, Sookmyung Women's University, Seoul, Republic of Korea; 4 World Class Institute, Korea Research Institute of Bioscience and Biotechnology, Ochang, Republic of Korea

## Abstract

Cystatin SN (CST1) is a specific inhibitor belonging to the cystatin superfamily that controls the proteolytic activities of cysteine proteases such as cathepsins. Our previous study showed that high CST1 expression enhances tumor metastasis and invasiveness in colorectal cancer. Recently, auranofin (AF), a gold(I)-containing thioredoxin reductase 1 (TrxR1) inhibitor, has been used clinically to treat rheumatoid arthritis. AF is a proteasome-associated deubiquitinase inhibitor and can act as an anti-tumor agent. In this study, we investigated whether CST1 expression induces autophagy and tumor cell survival. We also investigated the therapeutic effects of AF as an anti-tumor agent in colorectal cancer (CRC) cells. We found that CRC cells expressing high levels of CST1 undergo increased autophagy and exhibit chemotherapeutic resistance to AF-induced cell death, while those expressing low levels of CST1 are sensitive to AF. We also observed that knockdown of CST1 in high-CST1 CRC cells using *CST1*-specific small interfering RNAs attenuated autophagic activation and restored AF-induced cell mortality. Conversely, the overexpression of CST1 increased autophagy and viability in cells expressing low levels of CST1. Interestingly, high expression of CST1 attenuates AF-induced cell death by inhibiting intracellular reactive oxygen species (ROS) generation, as demonstrated by the fact that the blockage of ROS production reversed AF-induced cell death in CRC cells. In addition, upregulation of CST1 expression increased cellular glutathione reductase (GR) activity, reducing the cellular redox state and inducing autophagy in AF-treated CRC cells. These results suggest that high CST1 expression may be involved in autophagic induction and protects from AF-induced cell death by inhibition of ROS generation through the regulation of GR activity.

Cysteine proteases, such as cathepsins and papain, are proteolytic enzymes that are expressed widely in tissues and have numerous functions, including inflammatory tissue destruction, modulation of the immune response, tissue remodeling, and induction of monocyte and cancer cell migration.^1, 2^ Cathepsins are intracellular cysteine proteases that function in protein degradation in lysosomes^3^ and secretory granules. In various malignant tumors, cathepsins are overexpressed and localize to the invasive tumor margin.^4^ Previous clinical studies revealed that the upregulation of cysteine proteases is involved in various malignant tumors and that tumor growth and invasion can be regulated by inhibiting cysteine proteases. Cystatin superfamily proteins specifically inhibit the proteolytic activity of cysteine proteases.^5^ Cystatin SN is a secretory peptide encoded by the *CST1* gene that belongs to the type 2 cystatin superfamily.^6, 7, 8^ Previous studies reported that most type 2 cystatins are involved in tumor invasion and metastasis.^9, 10, 11, 12^ The upregulation of cystatin SN inhibits cathepsin and contributes to cell proliferation in gastric cancer.^9^ Cystatin SN was also identified as a novel tumor biomarker for colorectal cancer.^10, 11^ However, the relationship between cystatin SN expression and autophagy in colorectal cancer (CRC) has not yet been elucidated.

Autophagy is used by the cell to degrade misfolded proteins and damaged organelles^13^ and is known to protect against various forms of human disease.^14^ In cancer, however, autophagy contributes to both tumor suppression and tumor progression like a ‘double-edged sword'.^13, 14, 15, 16^ Autophagy-related genes (ATGs) regulate autophagy and are closely linked to cancer initiation and progression.^16^ However, an increasing number of reports consider autophagy to be an underlying mechanism of type II cell death.^13, 14^ Consequently, control of autophagy represents an important strategy in cancer treatment, and several autophagy-inhibiting or -promoting agents are already being used in anti-cancer therapies.^13, 14, 15, 16^

Reactive oxygen species (ROS) act as essential signaling messengers for various biological processes in both normal and cancer cells.^17^ The targeting of redox alterations represents another therapeutic strategy in cancer treatment.^17, 18, 19^ Moderate levels of ROS contribute to tumor development, promoting cancer survival signaling pathways such as proliferation, angiogenesis, and metastasis. However, excessive oxidative stress can cause DNA damage and an abnormal stress response, thus triggering cancer cell death.^18, 19^ Cellular ROS homeostasis is strictly controlled by balancing ROS-generating and scavenging systems such as thioredoxin (Trx), glutathione (GSH), superoxide dismutases (SOD1, SOD2, and SOD3) and catalase.^19^ Auranofin (AF) is a metal phophine complex that has been used for the clinical treatment of rheumatoid arthritis in pioneering studies conducted with gold(I) thiolate compounds.^20^ Recent studies suggested that AF acts as an inhibitor of thioredoxin reductase 1 (TrxR1), resulting in oxidative damage and modifications to cellular redox states, followed by over-production of ROS and apoptosis.^21, 22^ AF exerts a strong cytotoxic effect on several different types of neoplastic cells both *in vitro* and *in vivo*.^23, 24^ The cytotoxic activity of AF, along with its relatively safe profile in patients, warrants investigations into the therapeutic application of AF in cancer and other diseases.^25^ For example, AF is currently in clinical trials as a deubiquitinase inhibitor in hepatoma cancer cells.^26^

We previously reported that CST1 is significantly overexpressed in colorectal and gastric cancer, resulting in enhanced tumor cell growth and invasiveness.^9, 11^ In this study, we evaluated the role of CST1 upregulation on AF activation of autophagy-induced cell death in CRC cell lines and found that CST1 modulates AF-induced ROS production via thioredoxin reductase. Although CST1 expression has been examined in cancer proliferation, the role of CST1 in modulating the relationship between autophagy and ROS production under AF treatment has not been investigated. The mechanisms here elucidated indicate that CST1 may inhibit AF-induced CRC death *in vitro* by triggering ROS production, suggesting that CST1 may represent a potential target for colorectal cancer therapy.

## Results

### CST1 expression is elevated in CRC tissues and cell lines

**Figure 1:**
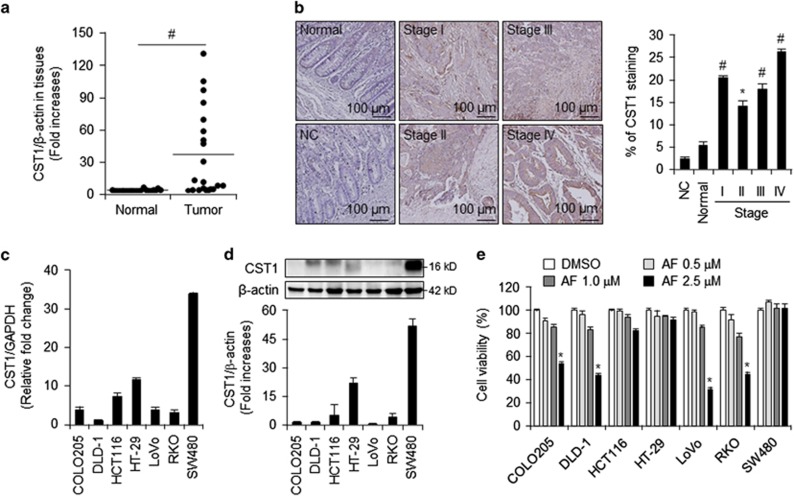
Upregulated expression of CST1 mRNA and protein in colon cancer tissues and effect of auranofin (AF) treatment on cell viability. (**a**) qPCR analysis of CST1 expression in 21 paired non-tumor colon (Normal) and colorectal tumor (Tumor) samples. (**b**) Immunohistochemistry assay with NC (negative control), anti-CST1 antibody and relative fold-change analysis were performed using tissue microarray chips consisting of 9 normal colons, 2 stage I, 11 stage II, 15 stage III, and 12 stage IV colorectal adenocarcinomas. (**c** and **d**) the basal level of CST1 expression in various colon cancer cell lines by qPCR (**c**) and western blot analysis, including relative band intensity (**d**). (**e**) WST-1 assay in colon cancer cell lines following treatment with various doses of AF for 24 h. All data shown are the mean±S.D. of three independent experiments. *P<0.05, ^#^*P<*0.01 *versus* control

To examine *CST1* mRNA levels in colorectal cancer tissues, we performed for real-time PCR and found that *CST1* mRNA expression was approximately 8-fold higher in colon cancer tissues than in normal tissues (Figure 1a). To investigate CRC stage-dependent expression of CST1, we conducted immunohistochemical (IHC) analysis of patient array chips. CST1 staining of tumor and paired normal tissues revealed elevated CST1 expression in CRC tissues compared with that in normal surrounding tissues (Figure 1b). When we analyzed a series of 59 patient samples of colon cancer tissues at various stages using by ImageJ (http://openwetware.org/wiki/Sean_Lauber:ImageJ-Threshold_Analysis), CST1 expression was higher in all stages of CRC tissues (14–26%) than in normal tissues (~5%), particularly in tumor stages I, III, and IV. We next investigated *CST1* mRNA and protein levels in the colon cancer cell lines COLO205, DLD-1, HCT-116, HT-29, LoVo, RKO, and SW480. *CST1* mRNA levels were elevated in most colon and CRC cell lines (Figure 1c). In HT-29 and SW480 cells in particular, CST1 was highly expressed at both the mRNA and protein levels (Figure 1d). To examine the relationship between CST1 expression and AF-induced cell death in colon cancer, we performed cell viability assays on the colon cancer cell lines following treatment with various doses of AF. Interestingly, the HCT-116, HT-29, and SW480 cell lines exhibited less cell mortality following treatment with 2.5 *μ*M AF than did the other cells lines (Figure 1e). These were also the three lines with the highest CST1 expression. These results suggest that CST1 expression is elevated in colon cancer tissues and cell lines and that the cytotoxicity of AF may be dependent upon CST1 expression.

### CST1 expression is associated with AF-induced apoptosis in colon cancer cell lines

**Figure 2:**
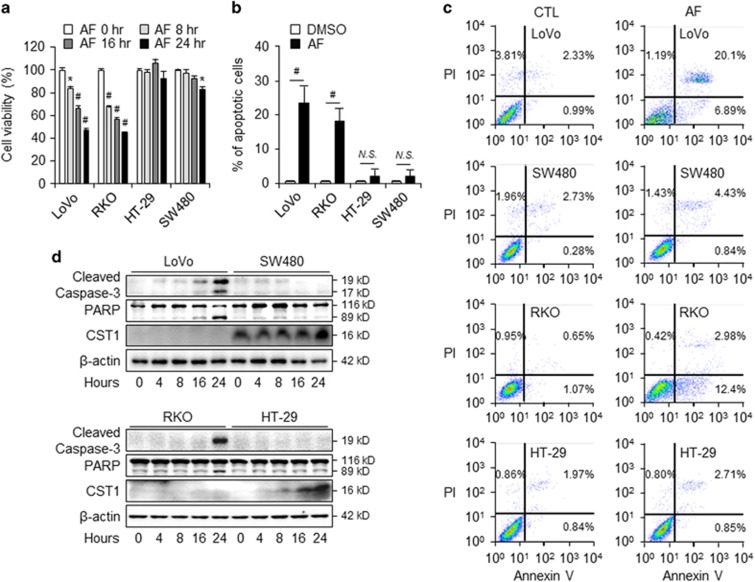
High-CST1 colon cancer cell lines are resistant to AF-induced cell death. (**a**) Cell viability assay in LoVo, RKO, HT-29, and SW480 cells following treatment with 2.5 *μ*M AF for various lengths of time. (**b**) Percentage of apoptotic cells as assessed by DAPI staining after treatment with 2.5 *μ*M AF for 24 h. (**c**) AF-induced cell death as measured by Annexin V-PI staining. Each cell was treated with 2.5 *μ*M AF for 16 h. (**d**) Cell death is dependent on CST1 expression, as analyzed by western blot using the apoptosis markers caspase-3, cleaved caspase-3, and PARP. All data shown are the mean±S.D. of three independent experiments. *P<0.05, ^#^*P<*0.01 *versus* control

To understand the relationship between CST1 expression and AF-induced cell death, we compared cell viability over time following AF treatment in the two cell lines with the lowest CST1 expression (LoVo and RKO) and those with the highest CST1 expression (HT-29 and SW480). Cells with low CST1 expression level exhibited increased levels of AF-induced cell mortality over time, while this pattern was not seen in cells with high CST1 expression (Figure 2a). We also conducted a nuclear fragmentation assay using DAPI staining. After AF treatment, while rates of DAPI-positive LoVo and RKO cells increased significantly (18–23%), there were few DAPI-positive HT-29 and SW480 cells (~3%) (Figure 2b). Because CST1 expression seemed to correlate with AF-induced cell death, we further assessed AF-induced cell death using an Annexin V-PI staining assay. Cells with low levels of CST1 expression (LoVo (26.99%) and RKO (15.38%)) increased total apoptosis ratios in both early (Annexin V-positive, PI-negative) and late (Annexin V-positive, PI-positive) apoptotic cells, while low ratios were observed in those with high levels of CST1 expression (HT-29 (3.56%) and SW480 (5.27%); Figure 2c). To confirm that these effects on AF-induced cell death were due to CST1 expression, we assessed protein levels of cleaved caspase-3 and cleaved PARP in cell lines with high and low CST1 expression after AF treatment for various lengths of time. As we expected, cells with low CST1 expression exhibited increased caspase-3 activation and PARP cleavage in response to AF treatment in a time-dependent manner. Again, this pattern was not observed in cells with high CST1 expression (Figure 2d). Therefore, we speculate that CST1 expression modulates AF-induced cell death in CRC cells.

### CST1 expression induces autophagy and attenuates AF-induced cell death in CRC cell lines

**Figure 3:**
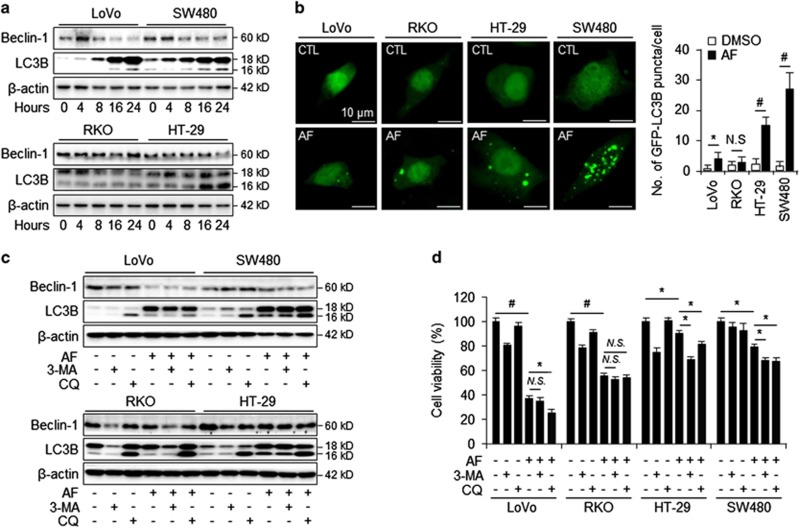
Autophagy inhibits AF-induced cell death. (**a**) Western blot of autophagic marker expression in LoVo, RKO, SW480, and HT-29 cells after treatment with 2.5 *μ*M AF for various lengths of time. (**b**) LoVo, RKO, HT-29, and SW480 cells were transfected with GFP-LC3B. Accumulation of GFP-LC3B punctae was detected after treatment with 2.5 *μ*M AF for 16 h. Graph shows the number of cells with GFP-LC3B punctae. (**c**) Confirmation of autophagy induction in LoVo, RKO, HT-29, and SW480 cells by autophagy flux experiment using western blot. Cells were treated with autophagy inhibitors 3-MA (5 mM), CQ (20 *μ*M), and 2.5 *μ*M AF for 24 h. (**d**) WST-1 cell viability assay showing effect of autophagy inhibitors on AF-induced cell death. All data shown are the mean±S.D. of three independent experiments. *P<0.05, ^#^*P<*0.01 *versus* control

To investigate whether CST1 expression induces autophagy following AF treatment, we performed western blot analysis to detect expression of the autophagy markers beclin-1 and LC3B in cells with high and low CST1 expression. While beclin-1 expression was not significantly different in low-CST1 (LoVo and RKO) and high-CST1 (HT-29 and SW480) cell lines (Figure 3a), LC3B form was elevated in high-CST1 cell lines following AF treatment. We next sought to examine morphological indices of autophagy in high- and low-CST1 cell lines under AF treatment by transfecting cells with green fluorescent protein (GFP)-LC3; the presence of GFP-LC3 puncta was then used as an indicator of autophagosome formation (Figure 3b). AF treatment resulted in increased formation of GFP-LC3 puncta in high-CST1 cell lines but not in low-CST1 cell lines.

To determine whether increases in LC3B activation correlated with increased autophagic flux during AF treatment, we repeated these experiments, treating cells with AF in the presence or absence of 3-methylaldehyde (3-MA), an inhibitor of phosphatidylinositol 3-kinase (PI3K) that blocks autophagosome formation, or chloroquine (CQ), an inhibitor of autophagosome-lysosome fusion. 3-MA treatment further reduced LC3B activation in high-CST1 cell lines under DMSO and AF treatments, indicative of autophagic activity, while CQ treatment further elevated the activation of LC3B-II form (Figure 3c). To confirm that the LC3B activation pattern reflects the autophagic processes, we used a WST-1 assay to determine whether the autophagy inhibitors affected AF-induced cell viability in high- and low-CST1 cell lines. Treatment of high-CST1 cell lines (HT-29 and SW480) with 3-MA and CQ significantly enhanced AF-induced cell death, while the presence of the autophagy inhibitors did not affect the mortality rate of AF-treated low-CST1 cell lines (LoVo and RKO; Figure 3d). We examined the presence of autophagosomes using cyto-ID autophagy assay (Supplementary figure S1A) Increased cyto-ID-positive cells were detected in AF-resistant cells HT-29 and SW480, corroborating that high-level CST1 expression is related with increased autophagy in AF-treated cells. Next, tandem fluorescent-tagged LC3 (mRFP–EGFP–LC3) construct is a convenient assay for monitoring autophagosomes (green) and autolysosomes (red) based on different pH stability of GFP and RFP fluorescent proteins, respectively. In CRC treated with AF, substantial increases in both yellow and red punctae were observed relative to the control, indicating that autophagic flux had increased in those cells (Supplementary figure S1B). These results support the hypothesis that CST1 expression increases autophagy, mediating AF-induced autophagic cell death in CRC cells.

### Increased CST1 expression inhibits AF-induced cell death by regulating autophagy

**Figure 4:**
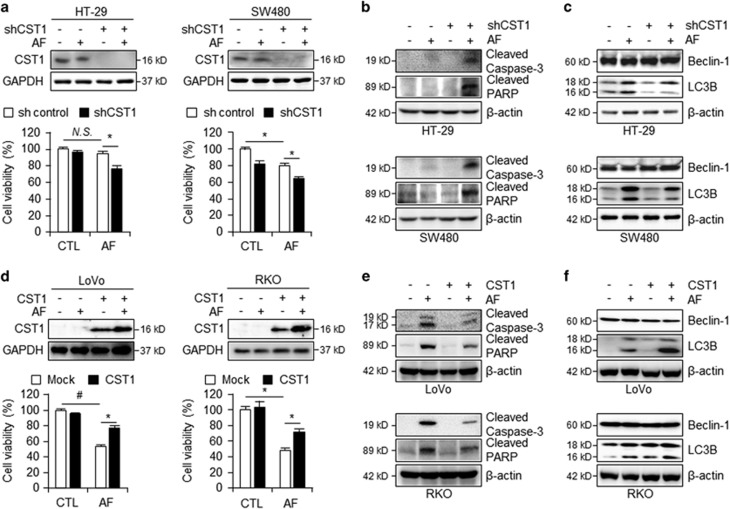
CST1 protects against AF-induced cell death through regulation of autophagy. (**a**) CST1 mRNA expression and WST-1 assay of HT-29 and SW480 shCST1 knockdown cell lines following treatment with 2.5 *μ*M AF for 24 h. (**b**) Western blot of apoptosis markers in shCST1 knockdown HT-29 and SW480 cells. (**c**) Western blot of autophagy markers was in shCST1 knockdown HT-29 and SW480 cells. (**d**) CST1 mRNA expression and WST-1 assay of LoVo and RKO CST1-overexpressing lines following treatment with 2.5 *μ*M AF for 24 h. (**e**) Western blot of apoptosis markers in CST1-overexpressing LoVo and RKO cells. (**f**) Western blot of autophagy markers in CST1-overexpressing LoVo and RKO cells. All data shown are the mean±S.D. of three independent experiments. *P<0.05, ^#^*P<*0.01 *versus* control

We hypothesized that the CST1 expression level is a critical factor in activation of autophagy and AF-induced autophagic cell death. To demonstrate that the loss/gain of CST1 function influences AF-mediated autophagic cell death, we induced small hairpin RNA-mediated CST1 (shCST1) knockdown in the high-CST1 lines HT-29 and SW480, and we transiently overexpressed CST1 with the pcDNA3.1-CST1 vector in the low-CST1 lines LoVo and RKO. As shown in Figure 4a, shCST1 knockdown in HT-29 and SW480 cells resulted in reductions in cell viability and CST1 expression after AF treatment. In contrast, CST1 overexpression in LoVo and RKO cells resulted in marked increases in cell viability and CST1 expression following AF treatment (Figure 4d). We also observed elevated levels of cleaved caspase-3 and cleaved PARP in shCST1 cell lines after AF treatment by western blot analysis (Figure 4b), while CST1 overexpression cells exhibited reduced cleaved caspase-3 and cleaved PARP levels (Figure 4e). Next, we investigated whether these changes in CST1 expression altered autophagic activation in AF-treated cells. Western blot analysis revealed that AF-treated shCST1 cells exhibited reduced activation of the autophagic marker LC3B (Figure 4c), while cells overexpressing CST1 exhibited elevated activation of LC3B (Figure 4f). These experiments indicate a link between CST1 expression and autophagy that appears to mediate AF-induced cell death in CRC.

### CST1 expression protects cells from AF-induced ROS production

**Figure 5:**
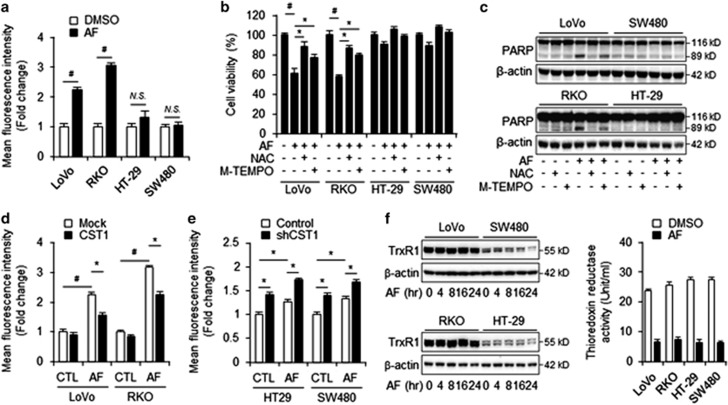
CST1 inhibits AF-induced cell death by regulating intracellular ROS. (**a**) CM-H_2_DCFDA was used for detection of AF-induced ROS by flow cytometry after 2 h of incubation with 2.5 *μ*M AF. (**b**) Effect of ROS scavengers NAC (1 mM) and M-TEMPO (100 *μ*M) on AF-induced cell death as measured by WST-1 assay. ROS scavengers were co-treated with 2.5 *μ*M AF for 24 h. (**c**) Western blot analysis to detect AF-induced cell death after co-incubation of AF and ROS scavengers for 24 h. (**d** and **e**) Intracellular ROS levels of CST1-overexpressing LoVo and RKO cells (**d**) and shCST1 knockdown HT-29 and SW480 cells (**e**) as measured by flow cytometry after 2 h of incubation with AF. (**f**) TrxR1 expression and activity in LoVo, RKO, HT-29, and SW480 cells after 12 h of incubation with AF as measured by western blot and TrxR1 assay kit. All data shown are the mean±S.D. of three independent experiments. *P<0.05, ^#^*P<*0.01 *versus* control

It has been previously shown that AF induces ROS production in various cancer cells by inhibiting TrxR1, and it is this excessive ROS production that is thought to be responsible for AF-induced cell death.^22, 23, 24^ We hypothesized that expression of the cysteine protease inhibitor CST1 contributes to the regulation of AF-induced ROS production and cell death in CRC cells. To assess the involvement of ROS production in the relationship between AF-induced cell death and CST1 expression, intracellular ROS levels were measured in high- and low-CST1 cells after AF treatment using an intracellular ROS assay kit. The molecule 2′,7′-dichlorodihydrofluorescein diacetate (H_2_DCFDA) is a cell-permeable, non-fluorescent probe that readily diffuses into cells, where it is oxidized by ROS to the highly fluorescent molecule 2′,7′-dichlorofluorescein (DCF). In low-CST1 LoVo and RKO cells, AF treatment increased the intracellular DCF fluorescence intensity by 2.3- and 3.2-fold, respectively, but AF treatment did not affect fluorescence in high-CST1 HT-29 and SW480 (Figure 5a). We next examined the impact of treatment with the antioxidant N-acetylcysteine (NAC) and the mitochondria-specific antioxidant M-TEMPO. Pre-treatment with NAC significantly inhibited AF-mediated ROS production and recovered cell viability in low-CST1 cells (LoVo and RKO; Figure 5b). However, pre-treatment with M-TEMPO failed to protect cells against AF-induced cell death, indicating that mitochondrial ROS are not involved in AF-mediated cell death. In addition, western blot analysis showed that NAC and M-TEMPO reduced AF-induced PARP cleavage in low-CST1 cells but did not significantly affect PARP cleavage in high-CST1 cells (Figure 5c).

To further explore the mechanisms by which expression of CST1 regulates AF-induced ROS production, we analyzed ROS production in shCST1 knockdown (HT-29 and SW480) and CST1-overexpressing (LoVo and RKO) cells. After AF treatment, LoVo and RKO cells which were transfected with mock vector exhibited high ROS production, while CST1-overexpressing LoVo and RKO cells exhibited reduced ROS generation (Figure 5d). In contrast, shCST1 knockdown of HT-29 and SW480 cells resulted in increased ROS production following AF treatment compared to that in mock-treated cells (Figure 5e). Interestingly, CST1 knockdown significantly increased the basal level of ROS production in HT-29 and SW480 cells.

As AF is known to be a TrxR1 inhibitor, we next investigated whether CST1 expression affects TrxR1 expression or activity following AF treatment. For this, we used western blot analysis to examine TrxR1 levels in high- and low-CST1 cell lines following treatment with AF for various lengths of time. While the length of AF treatment did not affect TrxR1 expression, low-CST1 cells (LoVo and RKO) exhibited higher basal levels of TrxR1 expression than high-CST1 cells (HT-29 and SW480) (Figure 5f, left). We also evaluated differences in the enzymatic activity of TrxR1 between low- and high-CST1 cells. TrxR1 activity was elevated in all colon cancer cell lines after AF treatment, and there were no differences in TrxR1 activity between high- and low-CST1 cells (Figure 5f, right). These results suggest that CST1 expression may attenuate AF-induced ROS generation, thereby protecting cells from ROS-induced cell death.

### CST1 regulates cellular GSH to maintain intracellular redox states

**Figure 6:**
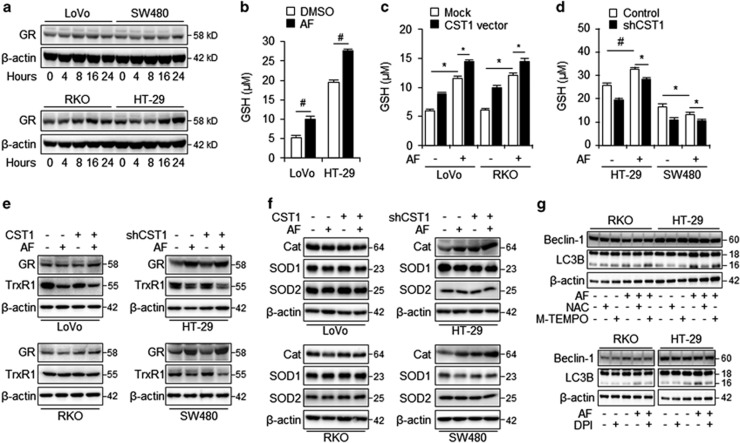
CST1 regulates cellular GSH to protect from AF-induced oxidative stress. (**a**) Western blot of GR levels in LoVo, RKO, HT-29, and SW480 cells following AF treatment for various lengths of time. (**b**) Cellular GSH levels in LoVo and HT-29 cells with or without AF treatment. (**c**) GSH levels in CST1-overexpressing LoVo and RKO cells after 12 h of incubation with AF. (**d**) GSH levels in shCST1 knockdown HT-29 and SW480 cells after 12 h of incubation with AF. (**e**) Western blot of GR and TrxR1 expression in CST1-overexpressing LoVo and RKO cells and shCST1 knockdown HT-29 and SW480 cells. (**f**) Western blot of catalase (Cat), SOD1, and SOD2 expression in CST1-overexpressing LoVo and RKO cells and shCST1 knockdown HT-29 and SW480 cells following AF treatment for 24 h. (**g**) Western blot of beclin-1 and LC3B expression in RKO and HT-29 cell lines following treatment with AF and ROS scavengers NAC (1 mM), M-TEMPO (100 *μ*M), or DPI (2.5 μM)

One important question is how CST1 attenuates AF-induced ROS generation. Along with the Trx system, the GSH system is one of the major intracellular disulfide-reducing systems.^27^ GSH acts as a major antioxidant within cells by maintaining tight control of the redox status.^28^ To determine whether CST1 regulates the GSH system, we used western blots to examine the expression levels of glutathione reductase (GR) in high- and low-CST1 cells following treatment with AF for various lengths of time. GR expression was not remarkably different in AF-sensitive and AF-resistant colon cancer cell lines (Figure 6a). We next measured total cellular GSH in low-CST1 (LoVo) and high-CST1 (HT-29) cells and found that AF treatment led to a greater increase in cellular GSH levels in high-CST1 cells compared to that in low-CST1 cells (Figure 6b). To further understand the mechanisms by which CST1 regulates AF-induced changes in cellular redox status, we evaluated levels of GR expression and cellular GSH following AF treatment in shCST1 and CST1-overexpressing cells (Figures 6c–f). While AF-treated LoVo and RKO cells transfected with mock vector still exhibited elevated cellular GSH levels, CST1-overexpressing LoVo and RKO cells exhibited even higher levels of GSH, both before and after AF treatment (Figure 6c). In contrast, while the GSH levels of mock-treated HT-29 and SW480 cells were not affected by AF treatment, shCST1 knockdown HT-29 and SW480 cells exhibited reduced cellular GSH levels after AF treatment (Figure 6d). Although cellular GSH levels were differentially regulated by CST1 expression, GR protein expression levels were unaltered by overexpression or knockdown of CST1 (Figure 6e).

Physiologically generated ROS are normally regulated by antioxidant enzymes, such as catalase (Cat) and superoxide dismutase (SOD).^29^ We further investigated whether CST1 expression regulates other antioxidant enzymes to maintain intracellular redox states. To do this, we examined the enzyme levels of the antioxidants catalase, SOD1, and SOD2 in shCST1 knockdown and CST1-overexpressing cells. The expression levels of catalase, SOD1, and SOD2 were not significantly altered by AF treatment or changes in CST1 expression in the CRC cells (Figure 6f). To confirm that ROS accumulation is associated with autophagic activation, high-CST1 (HT-29) and low-CST1 (RKO) cells were treated with one of three ROS scavengers: NAC, M-TEMPO, and diphenyleneiodonium (DPI), an NADPH oxidase inhibitor. While treatment of cells with NAC and DPI greatly reduced AF-induced LC3B-II conversion, treatment with the mitochondrial ROS scavenger M-TEMPO did not affect AF-induced LC3B-II conversion (Figure 6g). These findings suggest that total cellular GSH, a proxy for GR enzymatic activity, was elevated by high CST1 expression and may play a critical role in AF-induced cell death through NADPH oxidase-induced ROS generation.

## Discussion

Highly upregulated cystatins or cathepsins are implicated in early- and late-stage tumor cell progression, and altering the balance between cysteine proteases and cystatins may be clinically useful in combatting cancer.^30, 31^ Recent *in vitro* and *in vivo* studies have demonstrated that CST1 offers tumor cell death via pathways that are dependent on the inhibition of cysteine proteases such as cathepsin B or the induction of proliferation.^9^ CST1 expression is upregulated in CRC, contributing to colorectal tumorigenesis by neutralizing the inhibitory effect of cystatin C (CST3) on the proteolytic activity of cathepsin B.^11^ Here, we focused on the relationship between CST1 expression, the induction of autophagy, and AF-induced ROS production in CRC. We provided robust evidence to support the potential of CST1 as a therapeutic target and a biomarker for CRC. In this study, we revealed that CST1 mRNA and protein expression are elevated in colon cancer tissues and cell lines compared to levels in normal tissues. AF treatment inhibits the growth of CRC cells expressing low levels of CST1 in concentration- and time-dependent manners through simultaneous induction of apoptosis and autophagy. Induced apoptosis is demonstrated through chromatin condensation, upregulation of cleaved caspase-3, and accumulation of cleaved PARP. In contrast, high CST1 expression protects against AF-induced cell death, as the cytotoxicity of AF is dependent on CST1 expression.

Autophagy usually occurs in normal cells to maintain cellular turnover, clearance, and regeneration of new cellular components to restore balance in the system and is greatly increased in cells under pathological conditions that cause cell dysfunction such as trophic stress or nutritional deprivation, hypoxia, ischemia, endotoxin shock, and metabolic inhibition.^32, 33, 34, 35^ Autophagy induction may protect cells from apoptosis by eliminating damaged mitochondria and other organelles that have the potential to trigger apoptosis.^36, 37^ However, sustained over-activity or dysfunction of the autophagic pathway in pathologic states mediates a caspase independent form of cell death that shares certain features with apoptosis.^38, 39^ The importance of autophagy in aging and human disease, and the role of cystatin C in combating cell death via autophagic pathway has been investigated recently.^40, 41^ A similar paradigm for autophagic protein activation by cystatin C acting as a protective effector has been detailed by Tizon *et al.*^32^ This report demonstrated that the observed increase in the number of autophagosomes after exposure to cystatin C reflects induction of a fully functional autophagy via the mTOR pathway that includes competent proteolytic clearance of autophagy substrates by lysosomes. Recent *in vitro* and *in vivo* data have demonstrated that cystatin C plays protective roles via pathways that are dependent on inhibition of cysteine proteases, such as cathepsin B, or by induction of autophagy^42^ and protects neuronal cells against mutant copper-zinc superoxide dismutase-mediated toxicity.^43^ Our data suggest that the increase in CST1 expression in response to AF treatment induces autophagy as a chemotherapy-resistant mechanism. In this study, we demonstrated that high CST1 expression protects cells from AF-induced cell death and induces the redistribution of LC3 to vesicular profiles, with increased levels of immune-reactive LC3-II vesicles accompanied by increased conversion of LC3-I to LC3-II and autophagy flux. These observations demonstrate that CST1 expression induces autophagy as a general response to AF treatment in colorectal cancer. Moreover, knockdown of CST1 enhanced cell mortality in AF-treated high-CST1 cells. Conversely, overexpression of CST1 reduced mortality in cells with low basal CST1 expression. These experiments suggest that CST1 expression may regulate chemotherapy resistance in CRC cells.

Auranofin is a drug that has been approved for the treatment of rheumatoid arthritis.^44^ Recently, AF treatment was discovered to inhibit TrxR1 and induce ROS in cancer cells, which was associated with high *in vitro* and *in vivo* potency of AF against cancer cells.^45, 46^ AF has also been used as a proteasome-associated deubiquitinase inhibitor in hepatoma cancer cells.^47^ ROS are common initiators of the stress response, effectively leading to the induction of autophagic cell death.^48, 49^ ROS-generating agents such as H_2_O_2_ or 2-methoxyestradiol as well as chemical inhibitors of the mitochondrial electron transport chain can induce ROS production and autophagic cell death in both transformed and cancer cell lines.^50^ With the goal of potentially repurposing AF for the treatment of CRC, our study demonstrates the relationship between CST1-induced autophagy and AF-induced ROS production in CRC cells. In this vein, we also showed that low-CST1 cells generate high basal levels of ROS and that treatment with AF induces cell mortality via ROS production. Several lines of evidence demonstrate that ROS plays a vital role in AF-induced cell death in human CRC cells. Pre-treatment of cells with the ROS scavenger NAC or NADPH oxidase inhibitor DPI virtually provided significant protection from loss of viability with AF treatment, but not the mitochondrial ROS scavenger M-TEMPO. Measuring the cellular GSH levels will provide additional information regarding oxidative stress in these cells, as oxidative stress is often associated with a reduction in the total cellular GSH levels.^51^ In the current study, utilizing different approaches to modulate CST1 expression, we found that increased GR activity due to CST1 overexpression led to an inhibition of AF-induced autophagic cell death through ROS scavenging, whereas decreased GR activity due to shCST1 knockdown accentuated AF-induced autophagic cell death through increased ROS production. Therefore, the targeting of ROS represents an important strategy for cancer treatment. Additional investigations into the mechanisms of ROS-based treatments are required for further improving the efficacy and specificity of such cancer drugs.

These findings provide mechanistic evidence that AF is highly effective in suppressing the growth of human CRC cells via autophagic cell death, which is associated with ROS-dependent GR activation through CST1 expression. These findings contribute a novel pathway to our understanding of AF-induced cell death and may serve as a powerful basis for the design of AF-based pro-autophagic drugs for the treatment of colorectal cancer.

## Materials and Methods

### Cell culture and reagents

Colon cancer cell lines COLO205, DLD-1, HCT-116, HT-29, LoVo, RKO and SW480 were purchased from Korea Cell Line Bank (College of Medicine Seoul National University, Seoul, Korea), and cultured in RPMI1640 medium (Gibco, Waltham, MA, USA) supplemented with 10% fetal bovine serum (Gibco) and 1 × Antibiotic-Antimycotic (100 U/ml penicillin, 100 *μ*g/ml streptomycin, Fungizone 0.25 *μ*g/ml, Gibco) at 37 °C in a humidified incubator with 5% CO_2_. Auranofin and Mito-TEMPO were purchased from Enzo Life Sciences (Farmingdale, NY, USA). 3-MA, Chloroquine and NAC were from Sigma-Aldrich (St. Louis, MO, USA). Polyclonal Cystatin SN antibodies were generated by directly immunized BALB/c mice with human recombinant Cystatin SN antigen. The following antibodies were purchased by Santa Cruz Biotechnology (Santa Cruz, CA, USA): anti-APG5 (sc-33210), anti-BECN1 (sc-11427), anti-*β*-actin (sc-81178), anti-glutathione reductase (GR) (sc-32886), anti-thioredoxin reductase 1 (TrxR1) (sc-28321), anti-catalase (sc-50508), anti-superoxide dismutase 1 (SOD1) (sc-11407), anti-SOD2 (sc-30080). The following antibodies were obtained from Cell signaling Technology (Danvers, MA, USA): anti-cleaved Caspase-3 (#9661), anti-cleaved PARP (#5625), anti-PARP (#9542).

### Cell viability assay

Cell viability was assessed with the water-soluble tetrazolium salt (WST)-1 assay (Roche, Mannheim, Germany) according to the manufacturer's instructions. Briefly, colon cancer cell lines plated in 96-well plate (1 × 10^4^ cells/well) for 24 h were treated with DMSO or auranofin for the indicated time and doses. WST-1 reagent (10 *μ*l) was added to each well of a 96-well plate and incubated for 1 h. The conversion of WST-1 reagent into chromogenic formazan was quantitated with a multi-well spectrophotometer at OD_450_ (Molecular Devices, Sunnyvale, CA, USA). The autophagy inhibitors 3-methyladenine (3-MA; 5 mM) and chloroquine (CQ; 20 *μ*M) (both from Sigma, St. Louis, MO, USA) were added to serum-free medium for 24 h on day 1 after seeding.

### Immunohistochemistry (IHC) and human colon normal/cancer tissue sample

Human colon normal/cancer tissue array slides were purchased from SuperBioChips (SuperBioChips Laboratories, Seoul, Korea). Immunohistochemistry was performed using an anti-Cystatin SN antibody (Novus, Littleton, CO, USA) with CAS blocking solution (Invitrogen, Carlsbad, CA, USA) for overnight. The tissue array slides were then incubated with biotinylated secondary antibody for 1 h. After incubation, tissue array slides stained with DAB substrate kit (Vector Laboratories, Millbrae, CA, USA) at room temperature until suitable staining develops. Immunohistochemistry staining was performed as the manufacturer recommended (VECTASTAIN ABC KIT, Vector Laboratories).

### Quantitative PCR

The expression of human CST1 mRNA was determined by quantitative PCR (qPCR). Total RNA was extracted from colon cancer cell lines and normal/cancer tissue using TRIzol reagent (Invitrogen, Carlsbad, CA, USA) according to the manufacturer's instructions and cDNA synthesized from total RNA (5 *μ*g) using a reverse transcription kit (Promega, Madison, WI, USA). One microliter of cDNA was used for qPCR, and duplicate reactions were performed for each sample using a ABI Power SYBR green PCR Master Mix (Applied Biosystems, Warrington, UK) with the following primer sequences: *CST1* for qPCR (forward: 5′-ACA AGG CCA CCA AAG ATG AC-3′, reverse: 5′-GGGCTGGGACTTGGTACATA-3′), *GAPDH* (forward: 5′-CAATGACCCCTTCATTGACC-3′, reverse: 5′-TTGATTTTGGAGGGATCTCG-3′) on an ABI Step one plus instrument (Applied Biosystems). RNA quantity was normalized to GAPDH content, and gene expression was quantified according to the 2^−ΔCt^ method.

### Immunoblot analysis

All samples were lysed with radioimmunoprecipitation assay (RIPA) lysis buffer (50 mM Tris-HCl (pH 7.4), 150 mM NaCl, 1% NP40, 0.25% sodium deoxycholate, 1 mM phenylmethylsulfonylfluoride (PMSF), 1 mM sodium orthovanadate, 1x Sigma protease inhibitor cocktail) in ice for 30 min. Lysates were then quantified via Pierce BCA Protein Assay Kit (Thermo Scientific, Rockford, IL, USA). 10~50 *μ*g of protein was separated by 10–15% SDS-PAGE and then transferred onto Immobilon-P membrane (Millipore, Billerica, MA, USA). The membranes were blocked with 5% skim milk/TBS-T for 1 h and incubated with primary antibodies for overnight at 4 °C. After washed three times, the membranes were incubated with HRP-conjugated secondary antibodies for 40 min at room temperature. After 1.5 h washing, protein bands were visualized using Chemiluminescent HRP Substrate (Millipore).

### Transient transfection and establishment of stable cell lines

For CST1 overexpression, LoVo and RKO cell lines were seeded into six-well plate (1 × 10^6^cells/well). After 24 h, each cell line was transfected pcDNA3.1 mock or CST1 plasmid DNA using Lipofectamine 2000 (Invitrogen) according to the manufacturer's instructions. CST1 targeting shRNA expression cell lines were produced by lentivirus infection. CST1 shRNA was purchased from Sigma-Aldrich (Mission shRNA Plasmid DNA). Plasmid DNA was purified using Plasmid Midi Kit (Qiagen, Valencia, CA, USA) following the instructions of manufacturer. Purified shRNA plasmid DNA was transfected into Lenti-X 293T (Clontech, Mountain View, CA, USA) with Mission Lentiviral Packaging Mix (Sigma-Aldrich) using Lipofectimine 2000 (Invitrogen). The lentiviral particle harvested from culture media for 5 days, and then concentrated using Lenti-X™ Concentrator (Clontech) according to manufacturer's instructions. Concentrated lentiviral particles were infected with HT-29 and SW480 cell lines and selected by culturing in the presence of 4 *μ*g/ml puromycin (Sigma-Aldrich).

### Cyto-ID and DAPI staining assay

Nuclear morphological changes undergoing apoptosis were detected by staining with DAPI (Sigma-Aldrich). LoVo, RKO, HT-29 and SW480 cell lines were seed on glass coverslip in 6-well plate and treated with the presence or absence of auranofin for 12 h. Cells were washed three times with PBS and fixed by 4% paraformaldehyde for 30 min. Following wash with PBS, cells were incubated in a Cyto-ID (1:500) and DAPI solution (1 *μ*g/ml) for 30 min in the dark. After incubation, cells were washed then analyzed with a ZEISS LSM 510 META confocal microscope.

### Quantification of GFP-LC3 and GRP-RFP-LC3 puncta

LoVo, RKO, HT-29 and SW480 (2 × 10^5^ cells) were plated on glass coverslip. One day after, cells were transfected with pEGFP-LC3B, mCherry-RFP-GFP-LC3 vector by Lipofectamine 2000 (Invitrogen) and then incubated for 24 h at 37 °C. After transfection, 2.5 *μ*M Auranofin was treated for 12 h at 37 °C. Following drug treat, each cell was subjected to ZEISS LSM 510 META confocal microscope.

### Flow cytometry

Apoptotic cells were measured by FITC Annexin V Apoptosis Detection Kit I (BD Pharmingen, San diego, CA, USA) according to manufacturer's instructions. To measure total cellular H_2_O_2_, Cells were incubated with CM-H_2_DCFDA (Invitrogen) at final 5 *μ*M final concentration. Data were analyzed with a FACS Calibur flow cytometer (Becton Dickinson, San Jose, CA, USA).

### Determination of Thioredoxin reductase activity

Each cell was plated into 60 mm culture dishes and 1d after incubated with or without auranofin. Cells were washed with ice-cold PBS and lysed in dish. Cell lysate was centrifuged at 13,000 r.p.m. for 10 min and determine the protein concentration using Pierce BCA Protein Assay Kit (Thermo Scientific). Thioredoxin reductase activity was measured by Thioredoxin reductase assay kit (Sigma-Aldrich) according to manufacturer's instructions.

### Glutathione assay

Cellular glutathione level was measured using GSH-Glo Glutathione assay (Promega) according to manufacturer's instructions. In brief, 1 × 10^4^ cells were plated in white bottom 96-well plate. After indicated incubation, the culture media were removed carefully and three times washed with PBS. After wash, 100 *μ*l of prepared GSH-Glo reagent (Luciferin-NT substrate and Glutathione S-Transferase) added to each well of a 96-well plate and mix gently. After 30 min incubation at RT, 100 *μ*l of luciferin detection reagent added to each well of a 96-well plate and incubation for 15 min at RT. Following incubation, luminescence detected by The FilterMax F5 Multi-Mode Microplate Reader (Molecular Devices).

### Statistical analysis

Quantitative data are shown as means±S.D. and significance of statistical analysis was determined with two-tailed, unpaired Student's *t*-test. *P*-values <0.05 were considered significant.
