# Cystatin SN inhibits auranofin-induced cell death by autophagic induction and ROS regulation via glutathione reductase activity in colorectal cancer

**DOI:** 10.1038/cddis.2017.446

**Published:** 2017-09-14

**Authors:** Byung Moo Oh, Seon-Jin Lee, Hee Jun Cho, Yun Sun Park, Jong-Tae Kim, Suk Ran Yoon, Sang Chul Lee, Jong-Seok Lim, Bo-Yeon Kim, Yong-Kyung Choe, Hee Gu Lee

**Correction to:** *Cell Death and Disease* (2017) **8**, e2682; doi:10.1038/cddis.2017.100; published online 16 March 2017

In the version of this article originally published, the following affiliation for Sang Chul Lee was incorrectly listed as 3 instead of 1.

The correct affiliations are listed below:

Byung Moo Oh^1,2,5^, Seon-Jin Lee^1,2,5^, Hee Jun Cho^1^, Yun Sun Park^1^, Jong-Tae Kim^1^, Suk Ran Yoon^1^, Sang Chul Lee^1^, Jong-Seok Lim^3^, Bo-Yeon Kim^4^, Yong-Kyung Choe^1^ and Hee Gu Lee^1,2^

^1^Immunotherapy Convergence Research Center, Korea Research Institute of Bioscience and Biotechnology, Daejeon, Republic of Korea

^2^Department of Biomolecular Science, University of Science and Technology (UST), Daejeon, Republic of Korea

^3^Department of Biological Sciences, Sookmyung Women’s University, Seoul, Republic of Korea

^4^World Class Institute, Korea Research Institute of Bioscience and Biotechnology, Ochang, Republic of Korea

^5^These authors contributed equally to this work.

The corrected article appears online together with this erratum.

