# Supplementary material for: Cystatin SN inhibits auranofin-induced cell death by autophagic induction and ROS regulation via glutathione reductase activity in colorectal cancer
Source: Cell Death Dis. 2017 Mar 16;8(3):e2682–. doi: 10.1038/cddis.2017.100 (PMC5386512; doi:10.1038/cddis.2017.100)
Supplement: Supplementary Information [file cddis2017100x1.docx]

**Cystatin SN inhibits auranofin-induced cell death by autophagic induction and ROS regulation via glutathione reductase activity in colorectal cancer**

Running title: CST1 regulation of autophagy in colorectal cancer

Byung Moo Oh^1,2,*^, Seon-Jin Lee^1,2,*^, Hee Jun Cho^1^, Yun Sun Park^1^, Jong-Tae Kim^1^, Suk Ran Yoon^1^, Jong-Seok Lim^3^, Bo-Yeon Kim^4^, Yong-Kyung Choe^1^ & Hee Gu Lee^1,2,†^

Supplementary Figure S1

(A) Cyto-ID staining were used for detection of autophagy activation in AF-induced colorectal cancer cells. Images of Cyto-ID staining analyzed on random 100 cells of Cyto-ID positive staining cells and number of punctated cells also calculated; Scale bars= 10 μm (*p<0.05, # p<0.01). (B) Representative images of mRFP–GFP–LC3 punctae. Colocalization of GFP and red fluorescent protein (RFP), indicated by yellow dots in the overlapped GFP and RFP images, is visible in autophagosomes, whereas only RFP fluorescence, indicated by red punctae, is observed in autolysosomes. Scale bar = 10 μm.
