# Supplementary figures and images for: Cystatin SN inhibits auranofin-induced cell death by autophagic induction and ROS regulation via glutathione reductase activity in colorectal cancer
Source: Cell Death Dis. 2017 Mar 16;8(3):e2682–. doi: 10.1038/cddis.2017.100 (PMC5386512; doi:10.1038/cddis.2017.100)

A

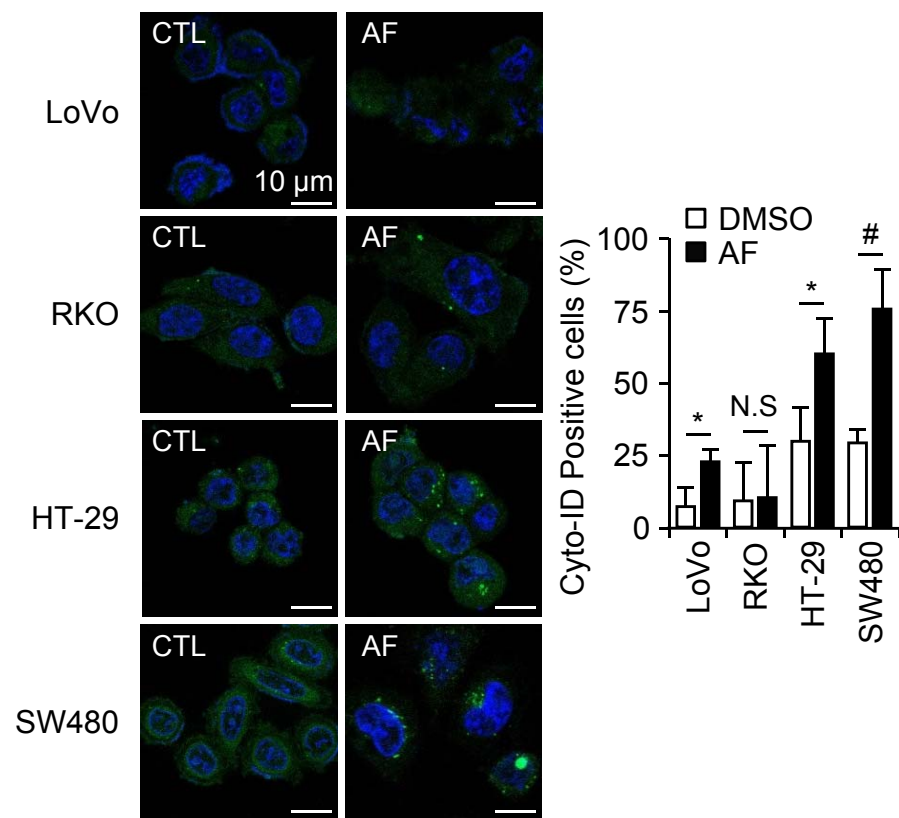

B

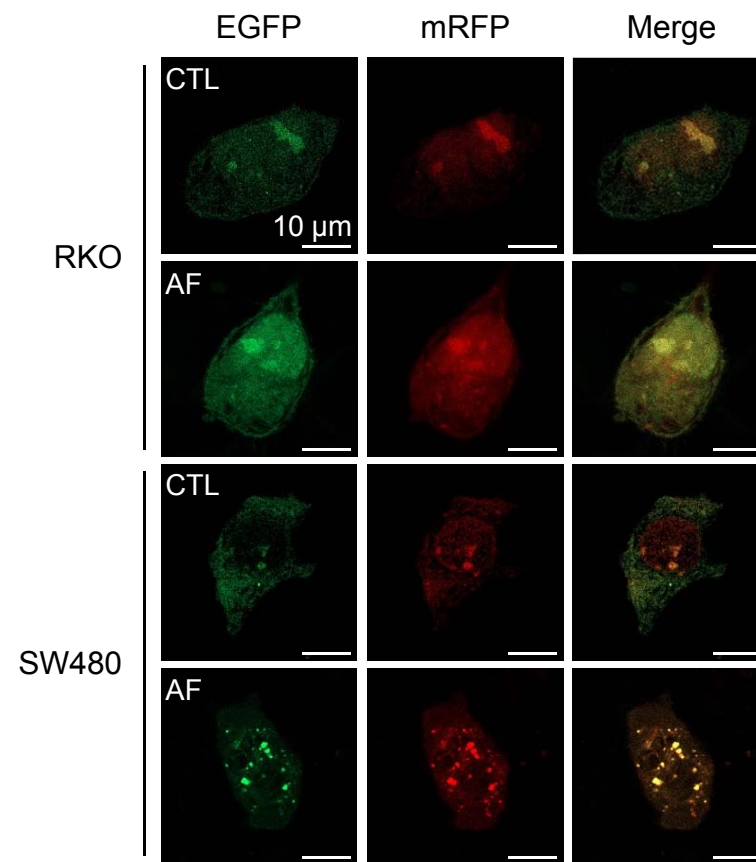

Supplementary Figure S1.

Supplement: Supplementary Figure 1 [file cddis2017100x2.pdf]
